# Transcriptomic and QTL Analysis of Seed Germination Vigor under Low Temperature in Weedy Rice WR04-6

**DOI:** 10.3390/plants12040871

**Published:** 2023-02-15

**Authors:** Wenjia Wang, Ruizhi Huang, Gengwei Wu, Jian Sun, Ying Zhu, Hua Wang

**Affiliations:** 1 State Key Laboratory for Managing Biotic and Chemical Threats to the Quality and Safety of Agro-Products, Institute of Virology and Biotechnology, Zhejiang Academy of Agricultural Sciences, Hangzhou 310021, China; 2 Rice Research Institute, Shenyang Agricultural University, Shenyang 110866, China

**Keywords:** weedy rice, seed germination, low temperature, RNA-seq, QTL

## Abstract

Low temperature is one of the major factors affecting rice germination, and low temperature germination (LTG) is an important agronomic trait. Although significant progress has been made in the study of rice LTG, the molecular mechanism of LTG remains poorly understood. To explore more rice LTG gene resources, we first demonstrated that weedy rice WR04-6 (*Oryza sativa* f. *spontanea*) had significantly higher LTG ability at 10 °C than the cultivated rice Qishanzhan (QSZ *Oryza sativa* L. ssp. *indica*). RNA-seq was used to investigate the gene expression of WR04-6 and QSZ at 10 °C for 10, 12 and 14 days after imbibition (DAI) of seed germination. The results of Gene Ontology (GO) enrichment and Kyoto Encyclopedia of Genes and Genomes (KEGG) enrichment revealed that the differentially expressed genes (DEGs) between WR04-6 and QSZ were mainly concentrated on the response to starch catabolic processes and the response to abscisic acid (ABA). This is consistent with the results of α-amylase activity, ABA and gibberellins (GA) treatment. A recombinant inbred line (RIL) population derived from a cross between WR04-6 and QSZ and its high-density SNP genetic map were used to detect quantitative trait loci (QTL) for LTG rates. The results showed that two new QTLs were located on chromosome 3 and chromosome 12. Combined with the mapped QTLs and RNA-seq DEGs, sixteen candidate genes potentially associated with LTG were identified. Validation of the expression of the candidates by qRT-PCR were consistent with the RNA-seq data. These results will enable us to understand the genetic basis of LTG in weedy rice and provide new genetic resources for the generation of rice germplasm with improved LTG.

## 1. Introduction

Rice production can be severely affected by temperature stresses [1,2]. Owing to its growth in tropical and temperate regions, rice is usually sensitive to chilling [3,4]. In many Asian countries, direct seeding of rice has become an inevitable trend in rice cultivation and development with labor shortage [5]. However, rice faces low-temperature stress during direct seeding in the Yangtze River basin and northern China, where temperatures during sowing usually range from 10 to 15 °C [6,7]. Low temperatures are especially detrimental from the germination to the reproduction stage and in most cases lead to reduced crop yields [8,9]. LTG is one of the most important traits required for direct seeding production of rice [10]. Improved LTG allows rice to have high germination vigor and stable seedlings under low-temperature production environments, which leads to yield stability [11]. Therefore, breeding rice germplasm with low-temperature tolerance and high germination capacity has become a focus for researchers.

Previous studies have shown that rice LTG is a quantitative trait controlled by multiple genes [12,13,14]. Given that rice originated in the tropics, genetic variation in cultivated rice germplasm is very limited in identifying cold tolerance genes and alleles [3]. The few examples of alleles associated with low-temperature tolerance, such as *qLTG3-1*, are mainly from cultivated rice [15,16]. *qLTG3-1* is the main QTL identified to date that controls the low-temperature germination rate in rice. In the process of seed germination, *qLTG3-1* is specifically expressed in the aleurone layer of the seed coat and the upper layer covering the coleoptile, which may increase seed germination potential at low temperatures by regulating the cell vacuolization of these tissues, thus causing their relaxation [15]. In addition, other candidate genes or QTLs related to LTG were also isolated, for example: *qLTG-1* [17], *qLTG-9* [18] and *qLTG_sRDP2-10a* [19]. Currently, most identified QTLs for LTG were based on the segregating populations from biparental crosses. In most cases, QTLs identified from different pairs of parents do not overlap, suggesting that more QTLs exist in natural populations. *OsSAP16* has been isolated as an LTG-related gene in a genome-wide association study (GWAS). Loss-of-function of *OsSAP16* reduced germination while overexpression of *OsSAP16* enhanced germination at low temperatures [20].

Rice domestication has undergone a long period of artificial selection [21,22], during which a large number of genes, including those associated with LTG, have been lost. However, these genes are preserved in weedy rice, such as the red pericarp gene, *Rc* [23,24]. Weedy rice is a major threat to paddy fields worldwide, with a morphology intermediate between wild and cultivated rice [25,26], and a unique semi-domestication process that allows it to possess a variety of characteristics of both wild and cultivated rice [27,28]. Weedy rice has rich genetic diversity and is an excellent rice germplasm resource [29,30]. Previous studies have shown that weedy rice is rich in nutrient elements and anthocyanins [24]. Weedy rice also has various stress resistances, owing to its unique geographical characteristics [31]. With low temperatures in early spring, seed germination is mainly affected by low temperatures, resulting in decreased germination [32,33]. Different from cultivated rice, direct seeding of weedy rice benefits from its stronger LTG ability. Furthermore, there is no reproductive isolation between weedy rice and cultivated rice. Therefore, weedy rice can be used as an excellent genetic resource for improving the LTG ability of cultivated rice.

WR04-6 is a weedy rice accession whose genome sequence has already been reported [29]. In order to explore more rice LTG genes, we investigated the difference in the LTG ability between WR04-6 and QSZ. Two low-temperature germination QTLs were detected with the RIL population derived from the progenies of WR04-6 × QSZ with which the genetic map contained 3581 SNPs. Using transcriptome analysis, 16 candidate genes were screened out. This study provides a new direction for the mining of LTG genes in rice.

## 2. Results

### 2.1. LTG Ability of WR04-6 and QSZ 

**Figure 1:**
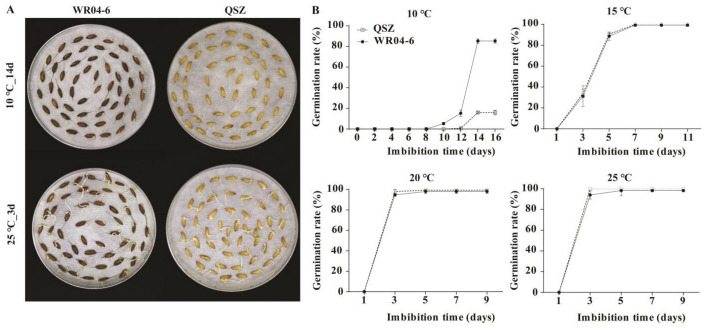
Low-temperature germinability of WR04-6 and QSZ. (**A**) Germination phenotype of WR04-6 and QSZ at 25 °C for 3 DAI and at 10 °C for 14 DAI. (**B**) Time course of germination rates at different temperatures. The modest square is QSZ. The solid circle is WR04-6. Three biological repeats were conducted. Error bars represent SD (*n* = 3).

The seed germination rates of WR04-6 and QSZ were tested under different temperatures (25 °C, 20 °C, 15 °C and 10 °C) to evaluate LTG. As shown in Figure 1, at 25 °C, 20 °C and 15 °C, there was no significant difference in the germination characteristics between the two accessions. At 25 °C and 20 °C, both WR04-6 and QSZ reached full germination at 3 DAI. At 15 °C, the seed germination speeds were slower than that at 25 °C and 20 °C, and both accessions reached full germination at 7 DAI. At 10 °C, the germination rates of WR04-6 and QSZ decreased significantly, and the germination times were delayed. At 10 °C, QSZ initiated germination at 12 DAI, whereas WR04-6 started germinating at 8 DAI. Both accessions reached their respective maximum germination rates (WR04-6, 85% and QSZ, 16%) at 14 DAI. These results indicated that weedy rice WR04-6 exhibited significantly higher LTG ability than cultivated rice QSZ (Figure 1).

### 2.2. RNA-seq Data Overview

RNA-seq analysis was performed on WR04-6 and QSZ germinated at 10 °C for 10, 12 and 14 DAI to elucidate the regulatory mechanisms of LTG vigor in WR04-6. Seventeen RNA libraries were established from three independent biological samples of two accessions. A total of 805 million raw reeds were generated. After eliminating low-quality reeds and performing quality checks, 785 million high-quality, clean reeds were obtained (Table S1). Clean reeds were mapped onto the rice genome https://phytozome.jgi.doe.gov/pz/portal.html#!info?alias=Org_Osativa (accessed on 27 November 2015). 

**Figure 2:**
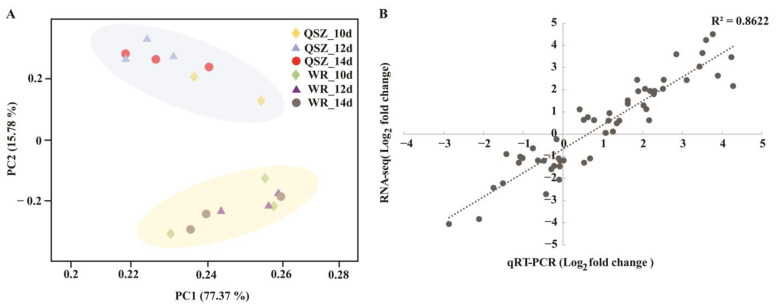
Evaluation of the reliability of RNA-seq data by PCA and qRT-PCR. (**A**) PCA of RNA-seq data of WR04-6 and QSZ under 10 °C for 10, 12 and 14 DAI. The X-axis represents the first principal component (PC1), and the Y-axis represents the second principal component (PC2). (**B**) Correlation between the log_2_ fold change in qRT-PCR (WR04-6 vs. QSZ) and RNA-seq (WR04-6 vs. QSZ).

Principal component analysis (PCA) showed that samples from WR04-6 and QSZ were clustered into two major groups (Figure 2A), indicating that there were great differences in the gene expression patterns of WR04-6 and QSZ during the LTG stage. Pearson’s correlation coefficients analysis also showed the RNA-seq data were highly reproducible and reliable (Figure S1). 

Gene expression was normalized using the Fragments Per Kilobase per Million (FPKM) method. DEGs were identified by pairwise comparison of the analyzed samples using the following criteria: *p*-value <  0.05 and |log_2_FC| ≥ 1 and q < 0.05. Using these criteria, a total of 9819 DEGs between WR04-6 and QSZ were detected (Figure S2). There are 6067 DEGs (3766 up-regulated and 2301 down-regulated) at 10 DAI, 6302 DEGs (4054 up-regulated and 2248 down-regulated) at 12 DAI and 5490 DEGs (3356 up-regulated and 2134 down-regulated) at 14 DAI in WR04-6 vs. QSZ. 

The gene expression levels of 13 randomly selected DEGs from each LTG stage were qualified by qRT-PCR to further evaluate the validity of DEGs characterized by transcriptomic analysis. The log_2_ foldchange value of DEGs quantified using qRT-PCR and RNA-seq were significantly correlated (R^2^ = 0.8622), confirming the reliability of RNA-seq expression data (Figure 2B).

### 2.3. Functional Classification of DEGs of WR04-6 vs. QSZ during LTG

**Figure 3:**
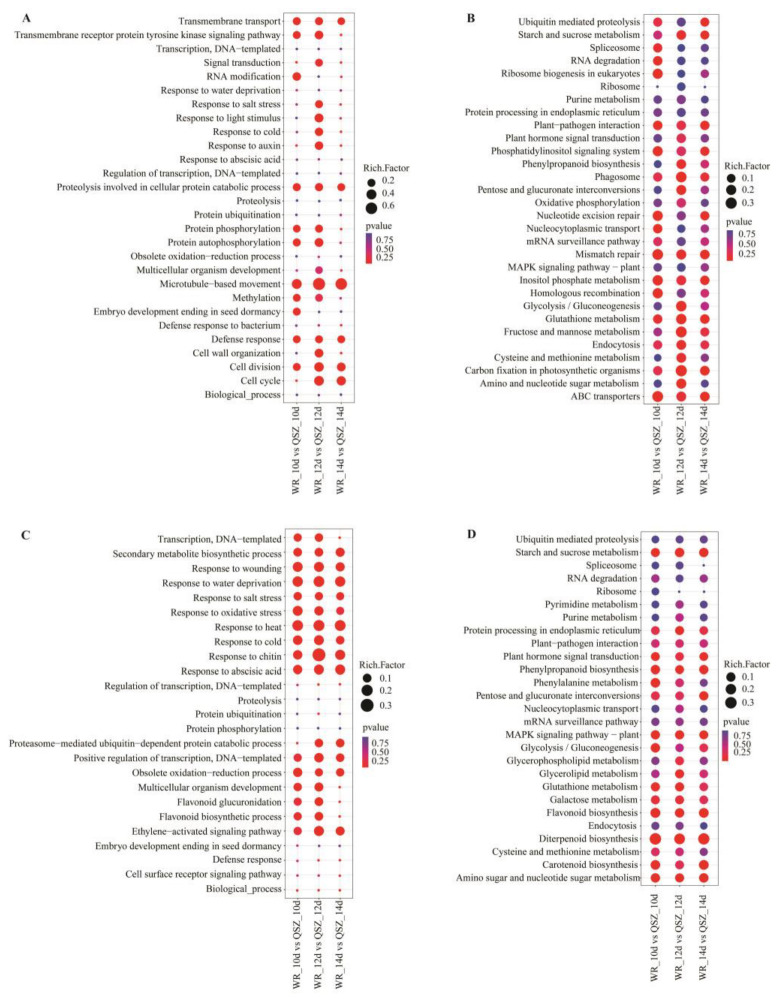
Enrichment analysis of DEGs between WR04-6 and QSZ at 10 °C. (**A**) Top 20 enriched GO-BP terms of the up-regulated DEGs (*p* ≤ 0.05). (**B**) Top 20 enriched KEGG pathways of the up-regulated DEGs (*p* ≤ 0.05). (**C**) Top 20 enriched GO-BP terms of the down-regulated DEGs (*p* ≤ 0.05). (**D**) Top 20 enriched KEGG pathways of the down-regulated DEGs (*p* ≤ 0.05).

GO and KEGG analyses were performed to predict the function of the identified DEGs. GO analysis of the DEGs revealed that the significantly enriched biological process was mainly associated with the oxidation-reduction process (GO:0055114), response to abscisic acid (GO:0009737), embryo development ending in seed dormancy (GO:0009793), response to salt (GO:0009651), signal transduction (GO:0007165), response to cold (GO:0009409), response to oxidative stress (GO:0006979), response to water deprivation (GO:0009414), response to auxin (GO:0009733) and flavonoid biosynthetic process (GO:0009813) (Figure 3A,C and Figure S3A,C,E). The KEGG pathway analysis revealed that the DEGs between WR04-6 and QSZ were involved in starch and sucrose metabolism (osa00500), Plant hormone signal transduction (osa04075), MAPK signaling pathway-plant (osa04016), Diterpenoid biosynthesis (osa00904), etc. (Figure 3B,D and Figure S3B,D,F). These results indicate that the DEGs of these terms play a vital role in regulating low-temperature tolerance during the germination stage.

**Figure 4:**
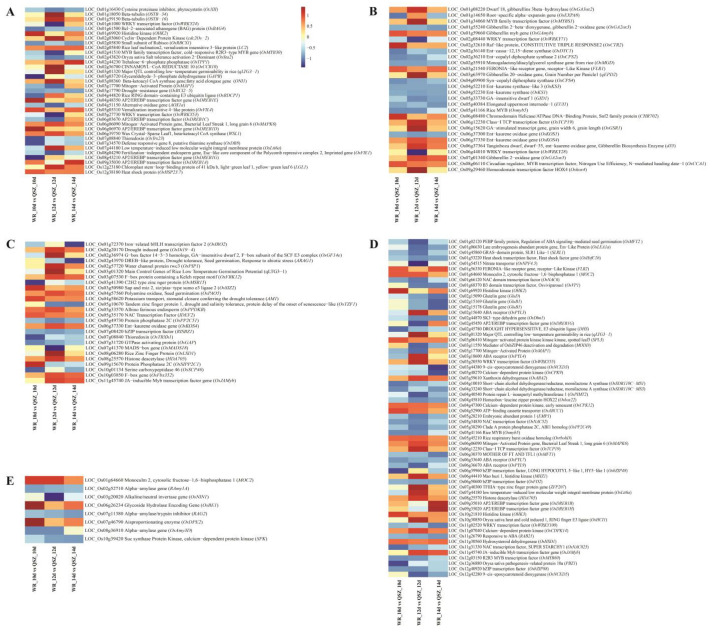
Heat maps of representative DEGs of different catalogs. (**A**) DEGs related to cold. (**B**) DEGs related to GA. (**C**) DEGs related to seed germination. (**D**) DEGs related to ABA. (**E**) DEGs related to α-amylase. The log_2_ fold change values of WR04-6 vs. QSZ at respective time points were used to create the heatmap. Red represents the up-regulated gene of WR04-6, and blue represents the down-regulated gene of WR04-6. The color depth indicates the degree of up and down.

Seed germination is a complex trait regulated by phytohormones and environmental conditions (such as temperature). In the present study, DEGs linked to starch and sucrose metabolism, phytohormone response and cold response were found by comparative transcriptome analysis of WR04-6 vs. QSZ during LTG (Figure 4A–D). The DEGs of seed germination were also identified by gene annotation (Figure 4E). 

ABA and GA are the major phytohormones that antagonistically control seed germination. In this research, 62 DEGs involved in ABA metabolism and signaling were detected, and 28 DEGs involved in GA metabolism and signaling were detected (Figure 4B,E). The ABA-related genes *OsMFT2*, *OsVP1*, *OsNAC52*, *RAB21* and *OsNCED5* were down-regulated in WR04-6. The GA-related genes *OsGA3ox2*, *OsGA2ox5*, *qEPD2*, *OsGAMYB* and *OsGSR1* were up-regulated in WR04-6.

Thirty-five DEGs involved in cold responses were found in the WR04-6 vs. QSZ comparison, among which the well-known cold stress tolerant related genes *OsLti6*, *OsDREB1D*, *OsDREB1A*, *OsHSP23.7* and *OsMAPK6* were up-regulated in WR04-6 (Figure 4A). It was found that *qLTG3-1*, a major control gene of LTG [15,16], was present in DEGs (Figure 4A,B,E). The RNA expression levels of *qLTG3-1* were significantly higher in WR04-6 than in QSZ at 10 °C for 12 and 14 DAI.

### 2.4. α-Amylase Activities of WR04-6 and QSZ during LTG

**Figure 5:**
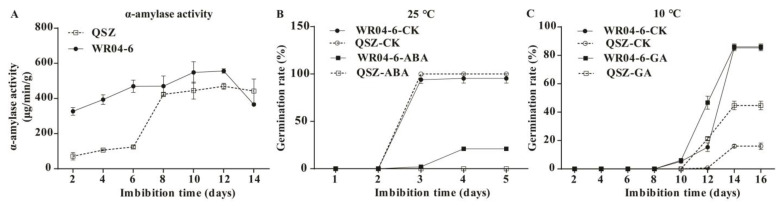
LTG-related index detection. (**A**) Time course of α-amylase activity of WR04-6 and QSZ germinated at 10 °C. (**B**) Germination behavior of WR04-6 and QSZ under 10 μM ABA treatment at 25 °C. (**C**) Germination behavior of WR04-6 and QSZ under 10 μM GA treatment at 10 °C. Error bars indicate SD (*n* = 3).

Starch is the main component of rice seed. During rice seed germination, α-amylase hydrolyzes starch into metabolizable sugars to provide energy and nourish the developing seedling. In this study, the KEGG pathway of “Starch and sucrose metabolism” was significantly enriched (Figure 3B,D), and several DEGs related to α-amylase were detected (Figure 4E). Therefore, we analyzed the dynamic changes of α-amylase activity in WR04-6 and QSZ during LTG. The results showed that both WR04-6 and QSZ represented a trend that first increased and then decreased (Figure 5A). Notably, α-amylase activity was higher in WR04-6 than in QSZ at the respective stages until 12 DAI, and the maximum α-amylase activity of WR04-6 was significantly higher than that of QSZ. Considering the importance of α-amylase in seed germination, the earlier induction of high level α-amylase activity of WR04-6 in the process of LTG contributed to the enhanced LTG ability of this accession.

### 2.5. Effect of ABA and GA on Seed Germination in WR04-6 and QSZ

The GO term “response to abscisic acid” was enriched in all three stages tested (Figure 3A,C and Figure S3). Genes related to GA metabolism and GA signaling were also detected in DEGs (Figure 4B). Thus, we determined the effects of ABA and GA on regulation of WR04-6 seed germination. The germination of WR04-6 and QSZ was inhibited and delayed with 10 mM ABA treatment at 25 °C. The germination rate of WR04-6 reached 21%, and that of QSZ was 0 (Figure 5B). WR04-6 showed more vigorous germinability than QSZ under ABA treatment conditions. In contrast, the germination of both accessions was identical with or without 10 μM GA treatment (data not shown). Both WR04-6 and QSZ did not germinate with the treatment of 10 μM ABA at 10 °C (data not shown). WR04-6 exhibited similar germination characteristics with or without 10 μM GA treatment. QSZ showed accelerated germination with 10 μM GA treatments (Figure 5C). It’s notable that at 10 °C, although the seed germination rate of QSZ increased from 16% to 44.6% with exogenous GA application, it was still significantly lower than that of WR04-6. The above results suggested that the better low-temperature germinability of WR04-6 was probably regulated via the ABA and/or GA pathways.

### 2.6. Identification of QTLs for LTG

**Figure 6:**
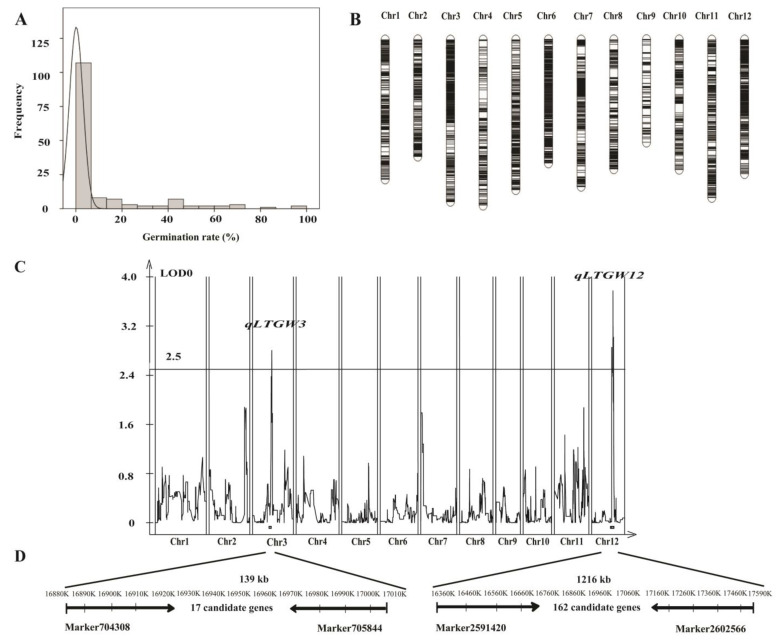
QTL mapping for the LTG. (**A**) The frequency distribution of LTG rates for the RIL population of WR04-6 × QSZ. (**B**) Genetic map of the RIL population constructed by WR04-6 × QSZ. Each black horizontal line is a marker. (**C**) QTL mapping of LTG in the RIL populations of WR04-6 × QSZ. (**D**) The QTL was narrowed down to a 139 kb region on chromosome 3 and a 1216 kb region on chromosome 12.

To isolate the candidate genes responsible for LTG, QTL mapping was carried out with an RIL population containing 137 individuals derived from WR04-6 and cultivated rice QSZ. First, we did a segregation analysis of LTG ability in this RIL population. The frequency distribution of LTG ability in the population did not show a clear bimodal pattern, suggesting that the phenotypic effect of this population is attributed to multiple genes (Figure 6A). Combined with a high-density genetic map containing 3581 SNP markers (Figure 6B), two QTLs with LTG were detected, namely *qLTGW3* (QTL for low-temperature germination in weedy rice) and *qLTGW12* (Figure 6C). 

Based on the reference genome of Nipponbare, *qLTGW3* was delimited by Marker704308 and Marker705844 on chromosome 3, in which the physical distance was about 139 kb and 17 genes harbored. Another QTL, *qLTGW12* was mapped between Marker2591420 and Marker2602566 on chromosome 12 with an interval of about 1216 kb, in which 162 genes existed (Figure 6D).

### 2.7. Candidate Gene Prediction

**Figure 7:**
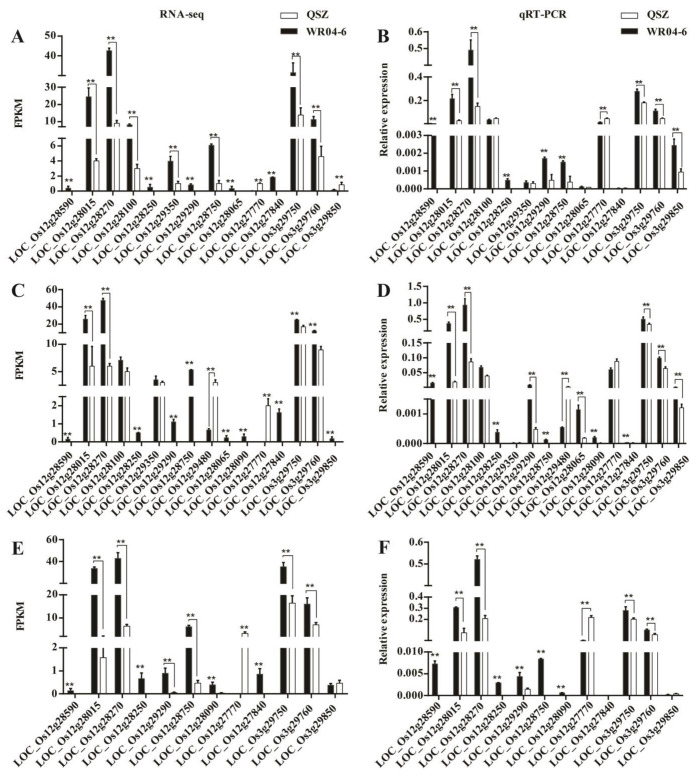
Expression level analysis of DEGs between WR04-6 and QSZ in the mapping intervals. (**A**,**B**) The expression level of DEGs at 10 °C for 10 DAI. (**C**,**D**) The expression level of DEGs at 10 °C for 12 DAI. (**E**,**F**) The expression level of DEGs at 10 °C for 14 DAI. Error bars represent SD (*n* = 3). The statistical significance was determined via Student’s *t*-test. **, *p* ≤ 0.01.

To further screen the candidate genes, we first compared the expression level of the genes in *qLTGW3* and *qLTGW12* with the DEGs of RNA-seq. RNA-seq data showed that 9 and 25 genes were expressed in *qLTGW3* and *qLTGW12* in the process of seed germination, respectively. Among them, there were 3 and 13 DEGs in *qLTGW3* and *qLTGW12*, respectively (Table S3). We tested 16 candidate genes by qRT-PCR. As shown in Figure 7, all genes exhibited significantly different expressions of WR04-6 vs. QSZ, similar to those found in RNA-seq results. Some of the genes, such as *LOC_Os12g28590* and *LOC_Os12g28250,* were expressed in WR04-6 at 10 °C for 10, 12 and 14 DAI, but not in QSZ. These data suggested that some of the candidate genes might be closely associated with LTG vigor of WR04-6.

## 3. Discussion

Previous studies on low temperature in rice have mainly focused on the seedling stage, less on LTG, and only in cultivated rice [15,17,18]. Weedy rice is a special type of rice that has no reproductive isolation with cultivated rice and can be freely hybridized, which makes it an excellent germplasm and genetic resource [30,34,35]. There have been some reports on the genes involved in stress resistance from weedy rice [31]. However, the molecular and genetic mechanisms of LTG in weedy rice are complex and require further investigation.

In this study, we first clarified that weedy rice WR04-6 has stronger LTG ability than QSZ at 10 °C. RNA-Seq was used to evaluate the transcriptomes of LTG between WR04-6 and QSZ. RNA-seq results revealed that 146 DEGs were enriched in starch catabolism, response to abscisic acid and response to cold pathways during the LTG process, which is possibly responsible for the LTG differences between WR04-6 and QSZ. α-amylase is the key enzyme for starch decomposition. The activity of α-amylase positively affects the ability of rice seed germination [36]. During seed germination, α-amylase gradually decomposed starch into glucose and maltose to provide energy for seed germination. In this study, the α-amylase activity of WR04-6 was higher than QSZ at 10 °C (Figure 5A), indicating that low temperature had a lower effect on seed germination in WR04-6 than QSZ. RNA-seq data also showed that a reported α-amylase suppressor gene *LOC_Os07g11380* (*RAG2*) in WR04-6 was significantly down-regulated (Figure 4E) [37]. These data suggested that the better LTG ability resulted from the higher activity of α-amylase in WR04-6.

Previous studies have shown that α-amylase gene expression is regulated by two phytohormones (ABA and GA), where GA is a positive regulator and ABA is a negative regulator. α-amylase is synthesized de novo during seed germination in the presence of endogenous GA from the embryo [38], suggesting that ABA and GA may play crucial roles in the regulation of seed germination [39,40]. The enrichment results showed that 88 DEGs of ABA and GA synthesis or signal transduction genes were involved in the germination process of WR04-6 at low temperature (Figure 3). OsMFT2 was found to provide a positive regulation for ABA-responsive genes and a negative regulation for rice seed germination by interacting with OsbZIP23/66/72 [41,42]. *OsNCED3* plays a role in regulating ABA content [43]. *Emp1* was shown at vegetative stages to be responsive to various abiotic stresses including drought, salt, coldness and the hormone ABA [44]. *OsWRKY71* encodes a transcription inhibitor in the process of GA signal transduction, which can also enhance the cold tolerance in plants [45,46]. At 10 °C, the expression level of *OsMFT2*, *OsNCED3*, *Emp1* and *OsWRKY71* in WR04-6 were significantly lower than that in QSZ. *OsPYL3* positively regulated the ABA response during seed germination. The overexpression of *OsPYL3* substantially improved drought and cold stress tolerance in rice [47]. In our study, the expression level of *OsPYL3* was higher in WR04-6 than in QSZ. These results indicated that ABA and/or GA signal transduction and synthesis gene expression of ABA or GA are affected by low-temperature conditions, and WR04-6 is endowed with greater cold tolerance to ensure normal seed germination. The expression level of these genes may be one of the reasons why WR04-6 has a stronger LTG ability than QSZ.

In addition to ABA and GA, the major gene responsible for LTG in rice, *qLTG3-1*, was enriched to seed germination and cold-related term. In the process of seed germination, *qLTG3-1* is specifically expressed in the aleurone layer of the seed coat and the upper germ layer covering the coleoptile, which may increase the germination potential of seeds at low temperature by regulating the cell vacuolation of these tissues [15,16]. RNA-seq results showed that *qLTG3-1* was a DEG, and its expression was higher in WR04-6 than that in QSZ (data not shown), but was not localized in the QTL mapping interval. This result indicated that *qLTG3-1* is a key gene responsible for the difference in LTG ability between WR04-6 and QSZ, but other genes still played an important role in the LTG of WR04-6. Other genes such as *OsPYL3*, *OsPAO5*, *OsMFT2*, *OsNCED3*, *Emp1* and *OsWRKY71* also played a significant role in LTG (Figure 5). 

Previous studies have shown that ABA and GA play key roles in the germination of *Arabidopsis* seeds. The expression of ABA- and GA-related genes in *Arabidopsis* is altered by low-temperature stress to regulate seed germination [48]. A large number of ABA, GA and α-amylase related DEGs between WR04-6 and QSZ were detected in RNA-seq, and changes in ABA and GA content affect the expression level of an α-amylase gene. Therefore, we speculate that WR04-6 may indirectly adjust α-amylase activity at low temperature by regulating the expression level of ABA- and GA-related genes to improve LTG ability.

LTG is a complex trait, as is evident from many QTLs identified from previous studies using biparental mapping. WR04-6 is the first weedy rice with an assembled genome [29], and a gene resistant to hypoxia germination (*OsGF14h*) has recently been isolated [31]. As a semi-domesticated species, weedy rice differs greatly from cultivated rice in the genome. It causes a stronger segregation phenomenon in the construction of genetic population, but also gives more potential genetic resources. In the present study, two QTLs (*qLTGW3* and *qLTGW12*) with LTG were first detected through QTL mapping from the RIL of WR04-6 × QSZ. Different from the traditional molecular marker mapping, the genetic map constructed by high-density SNP allows us to obtain a smaller QTL interval. New QTLs identified in this study indicate that more new genes contribute to variation in LTG in WR04-6 than previously detected.

Multigroup analysis is a common method in modern molecular biology. By combining RNA-seq and qRT-PCR analysis, 16 candidate genes were located in the two QTLs. Our qRT-PCR validation results are consistent with the RNA-seq results in the trend, but the relatively small value of gene expression level is due to the use of 2^−ΔCT^ value calculation. We found one of the candidate genes (*LOC_Os03g29760*) has been proven to be resistant to rice blast, bacterial blight, high salt and drought (Table S3). Based on the expression difference between WR04-6 and QSZ under low temperature, we speculate that *LOC_Os03g29760* may impart resistance to low-temperature stress. *LOC_Os12*g28590 and *LOC_Os12g28250* were expressed in WR04-6 at 10 °C, but not in QSZ (Figure 7). This obvious difference is consistent with its LTG phenotype, indicating that it may be related to LTG. *LOC_Os12g28100*, *LOC_ Os12g28250* and *LOC_Os12g29290* are disease resistance proteins, indicating that they are related to plant resistance. *LOC_Os12g28590* and *LOC_Os12g29350* are related to ATP protein and may participate in the pathway of starch hydrolysis to produce ATP during LTG (Table S3). Previous studies have shown that rice low-temperature tolerance genes include *CBF* (Calmodulin-binding transcription activator) and *MYB* (v-myb avian myeloblastosis viral oncogene homolog) families, etc. *CBF* responded quickly and transiently to low-temperature signals, while *MYB* responded slightly slowly to low-temperature stress [49,50]. The results showed that *LOC_Os12g28065*, *LOC_Os12g28100* and *LOC_Os12g29350* were only expressed at 10 and 12 DAI. *LOC_Os12g28090* was expressed at 12 and 14 DAI. *LOC_Os12g29480* was expressed at 12 DAI. The other candidate genes are expressed at 10, 12 and 14 DAI. This shows that these genes in WR04-6 respond to low temperatures at different stages. Further, there are many unknown genes and transposon proteins in the candidate genes, which will also be our potential target. Next, we will carry out functional verification around these key genes and explore their mechanisms. This endeavor will help us to improve the LTG ability of the cultivated rice in breeding programs.

## 4. Materials and Methods

### 4.1. Plant Materials

WR04-6 (weedy rice), was crossed with QSZ (cultivated rice) to produce the resultant F_1_ plants. One hundred and thirty-seven F_7_ lines (RILs) were developed from the resultant F_1_ plants using the single-seed descent method. The 137 RILs and their 2 parental lines were grown in the experimental base of Zhejiang Academy of Agricultural Sciences, Haining District, Hangzhou City, Zhejiang Province (120°12′ E, 30°16′ N). In the field experiments, plants were spaced at 30 cm × 10 cm. Mature seeds were collected for seed germination analysis.

### 4.2. Assay of Seed Germination

Fifty seeds from each accession (with three biological replications) were put in an oven at 50 °C for 48 h to break dormancy [18]. Then the seeds were placed on two layers of moistened filter paper in a 10 cm Petri dish and put into an incubator for temperature treatment. The seed germination test was conducted in the growth chamber at 10 °C, 15 °C, 20 °C and 25 °C under 12 h light/12 h day conditions. Seed germination was considered to have occurred when the epiblast was broken and the white embryo emerged. The germination rate was counted every 2 days within 14 DAI. All germination experiments were repeated three times in the same conditions.

### 4.3. RNA-seq

Low-temperature (10 °C) germinating seeds of WR04-6 and QSZ were collected at 10, 12 and 14 DAI, with 3 biological replications. Samples were immediately frozen in liquid nitrogen and stored at −80 °C until analyzed. The total RNA of the seeds was isolated and purified by TRIzol (Thermofisher, Waltham, MA, USA, 15596018). To control the quantity and purity of total RNA, NanoDrop ND 1000 (NanoDrop, Wilmington, DE, USA) was used, and to detect the integrity of RNA samples a Bioanalyzer 2100 (Agilent, CA, USA) was used. Finally, we used Illumina NovaseqTM 6000 (Illumina Inc., San Diego, CA, USA) to perform double-ended sequencing. The sequencing mode was PE150.

The Pearson correlation coefficient between samples and PCA were used to verify the repeatability among samples and exclude outliers. Significantly differentially expressed genes were identified based on a *p*-value of ≤0.05 and a log_2_ fold-change of (log_2_FC) ≥ 1. Ontology analyses of these genes were carried out by referring to GOseq http://geneontology.org (accessed on 24 February 2018) [51]. Pathway enrichment analyses were conducted using the KEGG database http://www.kegg.jp/kegg (accessed on 1 October 2017) [52].

### 4.4. RNA Extraction and qRT-PCR

The low-temperature (10 °C at 10, 12 and 14 DAI) germinating seeds of WR04-6 and QSZ used for qRT-PCR were the same samples used for RNA-seq. Total RNA was isolated individually from these germinating rice seeds using an E.Z.N.A ^®^Plant RNA Kit (Omega Bio-Tek, Norcross, GA, USA). The extracted 1µg RNA was reverse-transcripted to cDNA using a reverse transcription kit (Takara Bio Inc. Nojihigashi, Kusatsu, Shiga, Japan), and qRT-PCR was carried out in a total volume of 25 μL, containing 100 ng cDNA, 0.1 μM of each primer and 12.5 μL of 2X TB Green Premix Ex Taq (Tli RNaseH Plus) (Takara Bio Inc. Nojihigashi, Kusatsu, Shiga, Japan), with three biological replicates for each gene. The PCR program used for the qRT-PCR was as follows: a denaturation step (95 °C/30 s) followed by 40 cycles of 95 °C/5 s and 60 °C/30 s, and the final step of the Melt Curve was used for analysis to confirm the PCR product specificity. Data were collected in accordance with the CFX96 Real-Time PCR Detection System (Model No. CXF96^TM^ Optics Module, Bio-Rad Laboratories, Inc., Hercules, CA, USA). For validation of the RNA-seq data, thirteen genes scanned by RNA-seq were randomly selected, and the correlation between qRT-PCR results and RNA-seq data was analyzed to verify the accuracy of RNA-seq [53,54]. Primers were designed according to the gene ID using Primer5.0 and listed in Supplementary Table S2. The relative expression level data were analyzed using the 2^−ΔCT^ method, with *Actin* (*LOC_Os03g50885*) used as the internal control (Table S2). 

### 4.5. α-Amylase Activity Determination

The seed germination conditions were the same as for RNA-seq (10 °C). Germinating seeds were harvested at 2, 4, 6, 8, 10, 12 and 14 DAI for enzyme extraction with 3 biological replications. The α-amylase activity was determined by using a plant α-amylase (AMS) enzyme-linked immunosorbent assay kit (Suzhou Grace Biotechnology Co., Ltd., Suzhou, China), following the manufacturer’s instructions. We weighed 0.2 g of seeds after low-temperature treatment and extracted the bold matter with alcohol for determination.We tested α-amylase activity after the β-amylase was inactivated by heating at 70 °C. Starch was first converted to reducing sugar by catalyzing with α-amylase. Then, the reducing sugar was subjected to the reaction with 3, 5-dinitrosalicylic acid, which produced a brownish red product with maximum absorption at 540 nm. The α-amylase activity was detected by measuring the absorbance at 540 nm.

### 4.6. Characteristics of Seed Germination under ABA or GA Treatment

Seed germination tests treated by ABA or GA were performed as described above, but the water in the filter paper was replaced with 10 μM ABA or 10 μM GA solution [38,39,55]. ABA and GA temperature treatments were set at 10 °C and 25 °C. The number of germinated seeds was counted every day. 

### 4.7. QTL Mapping

To further explore the LTG genetic mechanism of WR04-6 and identify important LTG genes, an RIL population of 137 individuals was developed by single-seed descent from a cross between WR04-6 and QSZ. The germination rate of seeds from the RIL population was determined at the period (10 °C, 14 DAI) when the parental LTG ability was most significantly different. The population germination rate was measured simultaneously at 25 °C for 3 DAI as a control. The relative germination rate used for QTL was 10 °C for 14 DAI/ 25 °C for 3 DAI × 100% [19,20]. A total of 5,371 SNP markers from the genome assembly of WR04-6 and SLAF-seq of RIL [13,29,56] well-distributed in the whole genome were selected to screen both the parents WR04-6 and QSZ for polymorphism. SNPs that showed a good polymorphism in parents were used for genotyping of the RIL population. 3581 of 5371 SNPs were found to be highly polymorphic and were further used to construct linkage maps of the RIL population. The linkage map was constructed with MST map software for Linux [57] using Kosambi mapping distance and genotyping error detection. QTL mapping was performed by composite interval mapping (CIM) using the software WinQTL Cartographer v2.5. The experiment-wise threshold log of the odds ratio (LOD) scores for detection of QTL was calculated based on 1000 permutations at *p* ≤ 0.05 [58], and the LOD threshold value for declaring a QTL was set to 2.5. 

### 4.8. Statistical Analysis

All experiments were conducted with at least three biological replicates. Student’s *t*-tests were conducted using Microsoft Excel software to detect differences.

## 5. Conclusions

We demonstrated that WR04-6 has a stronger LTG ability than QSZ. The strong LTG of WR04-6 may be affected by ABA, GA and α-amylase and other related pathways. The *qLTG3-1* may play an important role in the LTG difference between WR04-6 and QSZ. Two new low-temperature germination QTLs were detected used the RIL population of WR04-6 × QSZ, and sixteen candidate genes were screened out. LTG genes in two new QTLs of WR04-6 can be utilized for the improvement of LTG in rice breeding programs, supporting the importance of utilizing natural variation for functional and breeding research in LTG.

## Data Availability

The datasets presented in this study can be found in online repositories. The names of the repository/repositories and accession number(s) can be found below: https://www.ncbi.nlm.nih.gov/ (accessed on 16 January 2023) GSE222929.

## Supplementary Materials

The following supporting information can be downloaded at: https://www.mdpi.com/article/10.3390/plants12040871/s1, Figure S1: The heatmap of Pearson correlation between samples; Figure S2: Summary of DEGs; Figure S3: Enrichment analyses of total DEGs (WR04-6 vs. QSZ); Table S1: Overview of sequencing data quality control; Table S2: qRT-PCR Primer. Table S3: List of candidate genes.
