# Supplementary material for: Transcriptomic and QTL Analysis of Seed Germination Vigor under Low Temperature in Weedy Rice WR04-6
Source: Plants (Basel). 2023 Feb 15;12(4):871. doi: 10.3390/plants12040871 (PMC9961040; doi:10.3390/plants12040871)
Supplement: Supplementary file 1 [file plants-12-00871-s001.zip › Table S1.pdf]

Table S1. Overview of sequencing data quality control.

Sample	Raw Data		Valid Data		Valid Ratio	Q20%	Q30%	GC content%
	Read	Base	Read	Base	(reads)			
QSZ_10d2	34344004	5.15	28565856	4.28	83.18	99.92	96.91	53
QSZ_10d3	35555362	5.33	32606178	4.89	91.71	99.94	97.09	53
QSZ_12d1	51916594	7.79	51458696	7.72	99.12	99.92	97.05	53
QSZ_12d2	48346492	7.25	47891396	7.18	99.06	99.92	96.99	54
QSZ_12d3	50291046	7.54	49861346	7.48	99.15	99.93	97.10	54
QSZ_14d1	50492880	7.57	50088782	7.51	99.20	99.96	96.71	54.50
QSZ_14d2	43699190	6.55	43288230	6.49	99.06	99.92	96.81	54
QSZ_14d3	41102810	6.17	40742252	6.11	99.12	99.91	97.01	53
WR_10d1	52667114	7.9	51589492	7.74	97.95	99.93	97.07	50
WR_10d2	53050928	7.96	52124358	7.82	98.25	99.92	97.06	50
WR_10d3	52241888	7.84	51037618	7.66	97.69	99.92	96.32	50
WR_12d1	52083670	7.81	51103902	7.67	98.12	99.93	96.54	50
WR_12d2	51578966	7.74	50319732	7.55	97.56	99.95	96.53	50.50
WR_12d3	53797474	8.07	52757812	7.91	98.07	99.93	96.46	50
WR_14d1	51391782	7.71	50517782	7.58	98.30	99.93	96.42	49.50
WR_14d2	43734482	6.56	42990932	6.45	98.30	99.94	95.88	50
WR_14d3	39272360	5.89	38578178	5.79	98.23	99.94	95.81	50
Total	805567042	121	785522542	118	97.18	99.93	96.69	51.68
