# Supplementary material for: Transcriptomic and QTL Analysis of Seed Germination Vigor under Low Temperature in Weedy Rice WR04-6
Source: Plants (Basel). 2023 Feb 15;12(4):871. doi: 10.3390/plants12040871 (PMC9961040; doi:10.3390/plants12040871)
Supplement: Supplementary file 1 [file plants-12-00871-s001.zip › Table S2.pdf]

Table S2. qRT-PCR Primer.

GeneID	Forward (5' to 3')	TM(℃)	Reverse (5' to 3')	TM(℃)	Amplicons length(bp)
LOC_Os03g50885(*Actin*)	CAACACCCCTGCTATGTACG	60	CATCACCAGAGTCCAACACAA	60	92
LOC_Os02g07160	AGGAGACAGGCCAACCTTTT	60.1	GAACTGTAGGGGCTTGCTTG	59.9	186
LOC_Os03g29730	AAAGTTGAGGCGGTGGTATG	60.1	ATTCTGGCCCTTTTCAGGAT	59.9	177
LOC_Os03g29750	CTGCTGGATACGGTGGTTTT	60	TTGGCCCATCATGTTGTAGA	60	218
LOC_Os03g29760	TGCTGTTGCAAGAAATGGAG	60	GTGAGGTGTGTTGTCCGTTG	59.9	201
LOC_Os03g29850	GAGGGTAAGCTGAGCAGCAC	60.8	AAGACGGAGTGGAAGCAGAG	60	155
LOC_Os04g41830	ACCACAGCTCCTCGAAGAAA	60	CTGTTGGACCTCCAATTGCT	60.1	168
LOC_Os04g43200	GGGCTTCAACGTGATTGTTT	60	TAAACCTTCCCTCGTTGTCG	60.1	173
LOC_Os05g35690	TCCACATGGACAAGCTTACG	59.7	GGTGGTCATGTTGGCGTAG	60	181
LOC_Os06g02490	TTGCCTGACACAATTTCCAA	60.1	TGTAATCGGCCATAGCTTCC	60.1	178
LOC_Os07g29760	GACCGACGTCAAGATGCAG	60.3	GTCGCACTCCGTCTTCTTCT	59.9	222
LOC_Os08g10570	GGAAGCAATCGAACAAAGGA	60.2	TGTTGCAATACCACCAGCAT	60	155
LOC_Os10g30450	AAGGAGCCCAAAGAGGAGAC	59.8	GCTGAGCTTGTCCTTGAGGT	59.6	195
LOC_Os12g27770	ATGGGCACATTGACTCATCA	59.9	CTCACGCTCCCTTTCTTCAC	60	173
LOC_Os12g27840	GCGGAATCGAAGGAGAAGTA	59.4	CATCGTGATTCCCCATCTGT	60.7	205
LOC_Os12g28015	AGATCCGACGGGAGGAAG	60.2	ATTTGCTGGCAAACAAAAGG	60.1	187
LOC_Os12g28065	CGATGGTCAGCTCCTTCTTC	60	TGCGATGAACAATACGTGCT	59.3	210
LOC_Os12g28090	GAAGAAGGAGCTGACCATCG	60	TCACCATCATCCACCAGAGA	60	204
LOC_Os12g28100	ACTGCTCAAGCTACCCCTGA	60	GGTGCTCGATGCCAATAAGT	60.1	208

LOC_Os12g28250	GGTTTCCAGTTCGTCG GTTA	60	CATTGTGAGCTTGTCG CACT	60	175
LOC_Os12g28270	GTTGGGGTTTGCTCCA ATTA	59.8	AGAATGAGCAGCCAGC AAAT	60	166
LOC_Os12g28590	AGGAAGAGAGACACG GACGA	60	CATATATCCGCCCCAT TTTG	60	154
LOC_Os12g28750	GTACACGGTGGACGTG ATGA	60.5	TGGTGTACTCGAAGGA GACG	58.9	209
LOC_Os12g29290	CTGCCCAATGTTGACA TCAC	60	ACATTGGGCAGGCACT AATC	60.1	188
LOC_Os12g29350	CAAGGACCTCCATTTC CTCA	60	CCATTGCTGAAGATGT GTGG	60.2	215
LOC_Os12g29480	AGGAAGACGACAACG ACCAC	60.2	GACCACGTCCACCACC TC	59.9	194
