# Supplementary material for: Transcriptomic and QTL Analysis of Seed Germination Vigor under Low Temperature in Weedy Rice WR04-6
Source: Plants (Basel). 2023 Feb 15;12(4):871. doi: 10.3390/plants12040871 (PMC9961040; doi:10.3390/plants12040871)
Supplement: Supplementary file 1 [file plants-12-00871-s001.zip › Table S3.pdf]

Table S3. List of candidate genes.

GeneID	Description	Name
LOC_Os03g29750	Expressed protein	
LOC_Os03g29760	Heme activator protein gene, nuclear factor Y subunit A, CCAAT-box-binding transcription factor	*OsHAP2E*,*OsNF-YA2*
LOC_Os03g29850	ZRT- and IRT-like protein, metal cation transporter	*OsZIP2*
LOC_Os12g27770	Transposon protein, putative, CACTA, En/Spm sub-class, expressed	
LOC_Os12g27840	Expressed protein	
LOC_Os12g28015	Expressed protein	
LOC_Os12g28065	Expressed protein	
LOC_Os12g28090	Expressed protein	
LOC_Os12g28100	NBS-LRR disease resistance protein, putative, expressed	
LOC_Os12g28250	Disease resistance protein RPM1, putative, expressed	
LOC_Os12g28270	Amidohydrolase, putative, expressed	
LOC_Os12g28590	ATPase 2, putative, expressed	
LOC_Os12g28750	Homologues of tapetum determinant1	*OsTDL1A, MIL2*
LOC_Os12g29290	Disease resistance protein RGA3, putative, expressed	
LOC_Os12g29350	ATP binding protein, putative, expressed	
LOC_Os12g29480	SAM domain containing protein, putative, expressed	
