# Supplementary figures and images for: Transcriptomic and QTL Analysis of Seed Germination Vigor under Low Temperature in Weedy Rice WR04-6
Source: Plants (Basel). 2023 Feb 15;12(4):871. doi: 10.3390/plants12040871 (PMC9961040; doi:10.3390/plants12040871)

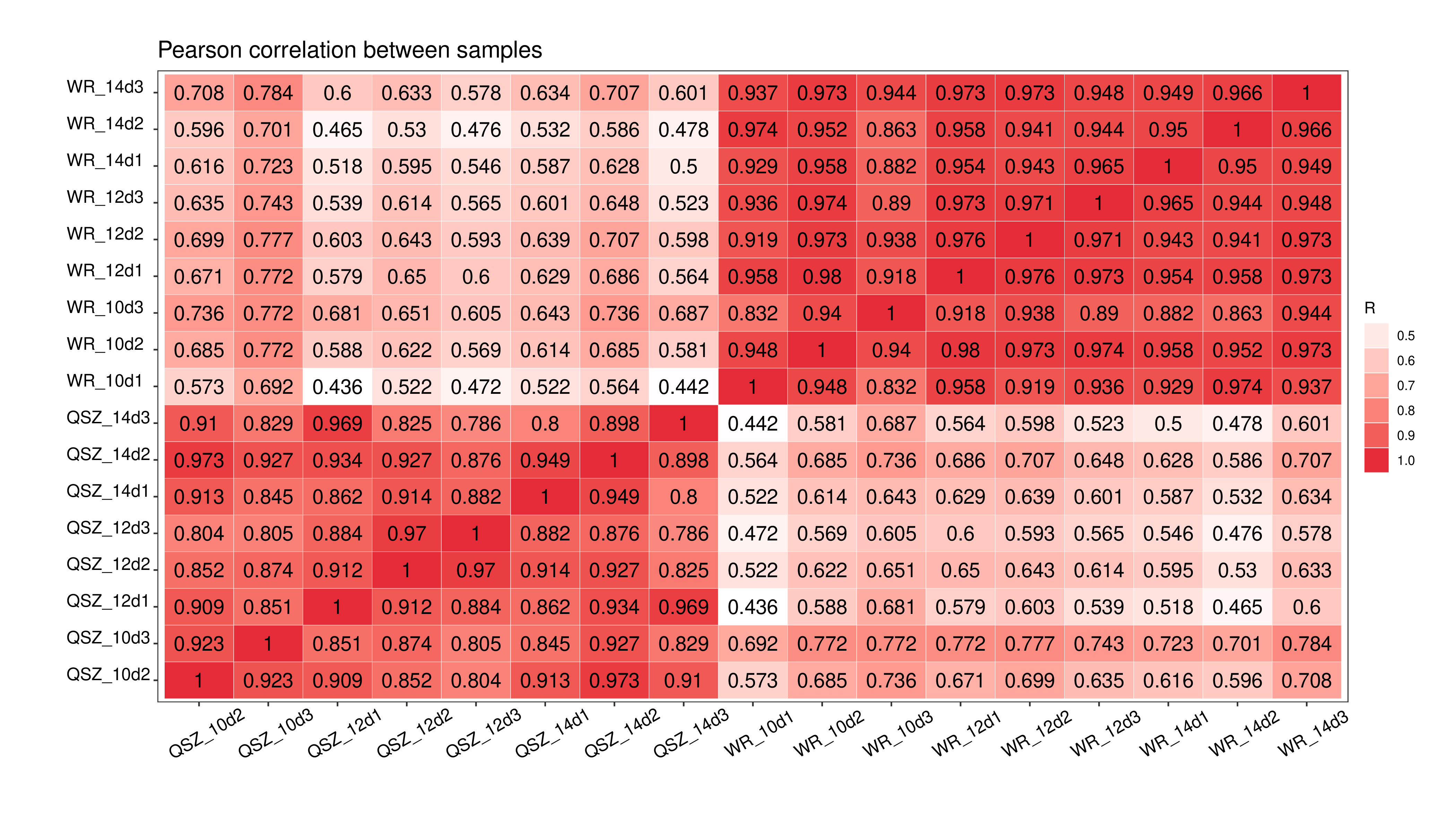

Supplement: Supplementary file 1 [file plants-12-00871-s001.zip › Figure S1. The heatmap of Pearson correlation between samples.png]

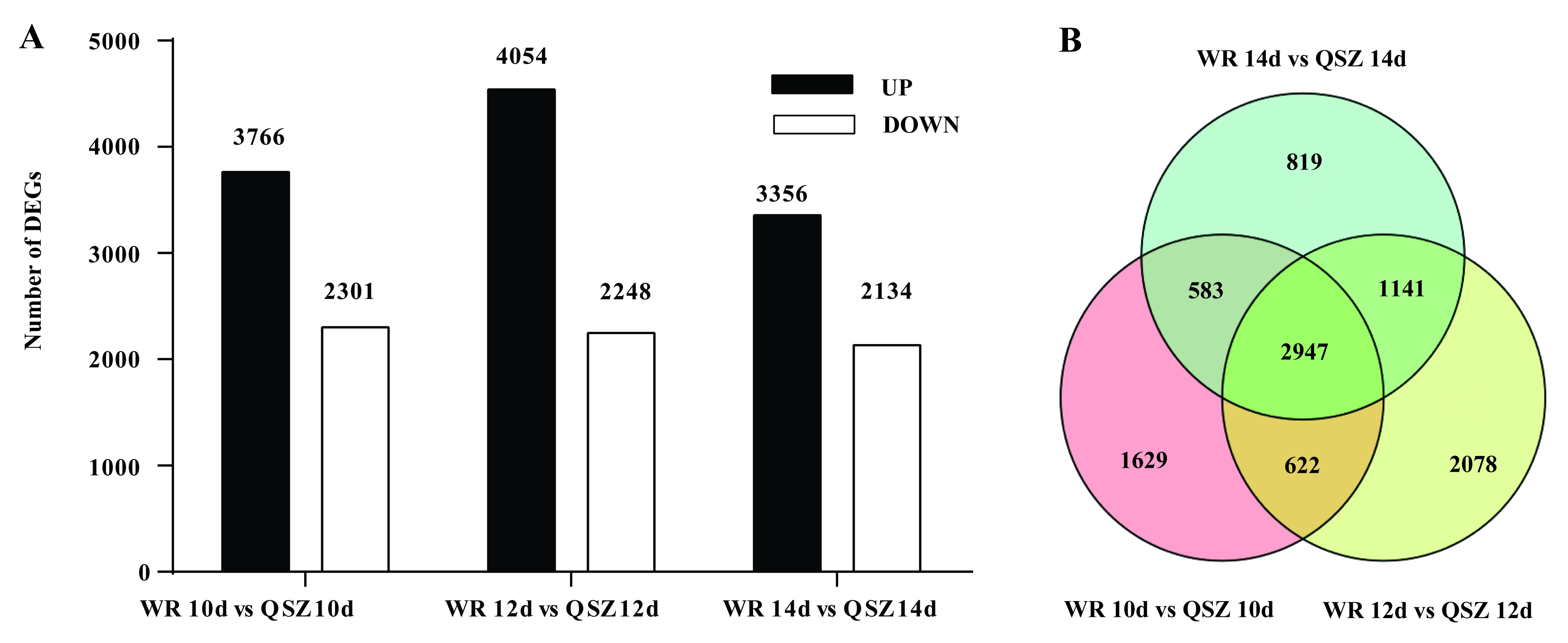

Supplement: Supplementary file 1 [file plants-12-00871-s001.zip › Figure S2. Summary of DEGs.jpg]

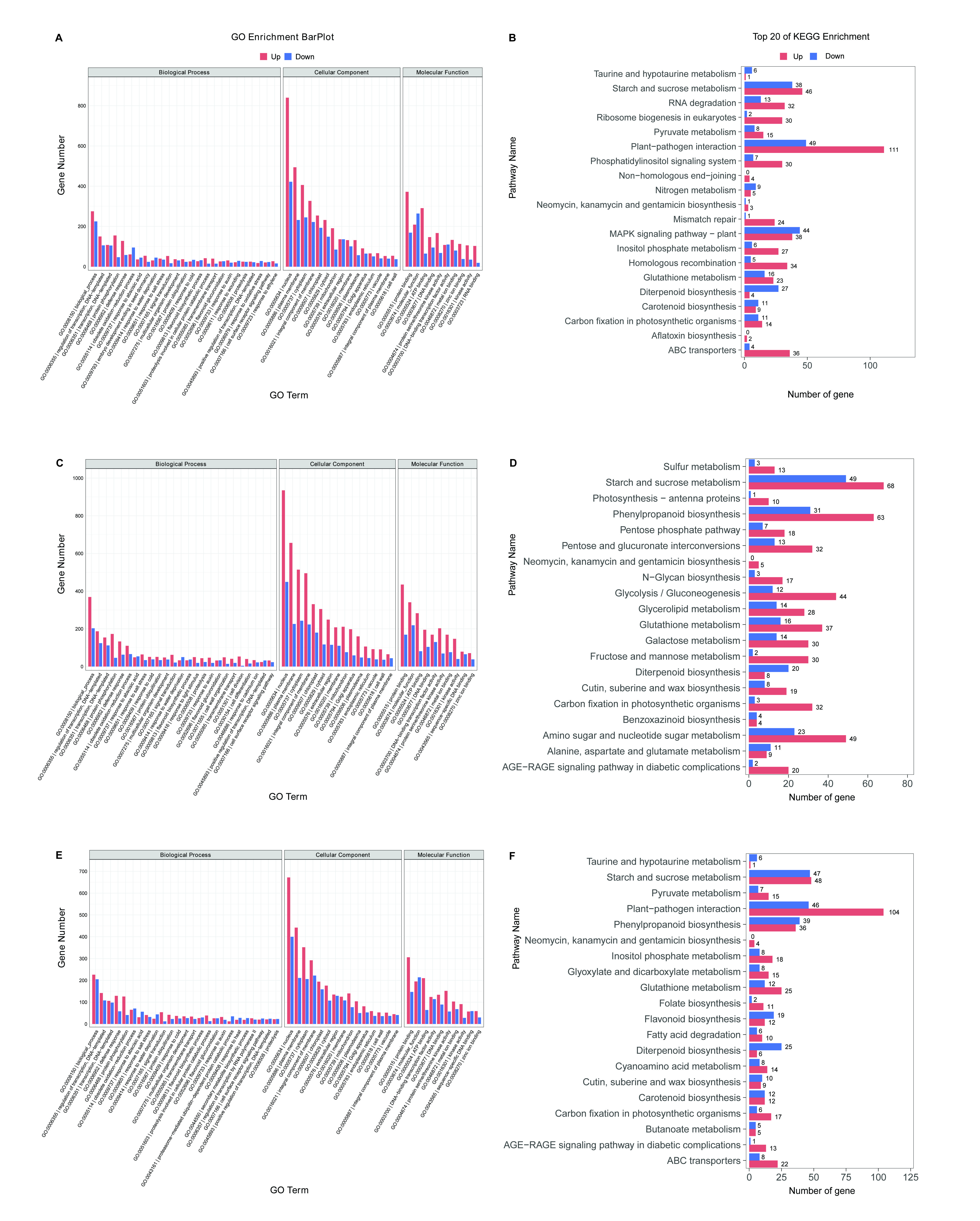

Supplement: Supplementary file 1 [file plants-12-00871-s001.zip › Figure S3. Enrichment analyses of total DEGs (WR04-6 vs. QSZ).jpg]
